# Patient responsiveness to a differential deductible: empirical results from The Netherlands

**DOI:** 10.1007/s10198-018-1014-y

**Published:** 2018-12-11

**Authors:** Stéphanie A. van der Geest, Marco Varkevisser

**Affiliations:** Erasmus School of Health Policy & Management (ESHPM), Erasmus University Rotterdam, P.O. Box 1738, 3000 DR Rotterdam, The Netherlands

**Keywords:** Patient channeling, Preferred providers, Tiered networks, Patient choice, I11, I13, D12, C25

## Abstract

Health insurers may use financial incentives to encourage their enrollees to choose preferred providers for medical treatment. Empirical evidence whether differences in cost-sharing rates across providers affects patient choice behavior is, especially from Europe, limited. This paper examines the effect of a differential deductible to steer patient provider choice in a Dutch regional market for varicose veins treatment. Using individual patients’ choice data and information about their out-of-pocket payments covering the year of the experiment and 1 year before, we estimate a conditional logit model that explicitly controls for pre-existing patient preferences. Our results suggest that in this natural experiment designating preferred providers and waiving the deductible for enrollees using these providers significantly influenced patient choice. The average cross-price elasticity of demand is found to be 0.02, indicating that patient responsiveness to the cost-sharing differential itself was low. Unlike fixed cost-sharing differences, the deductible exemption was conditional on the patient’s other medical expenses occurring in the policy year. The differential deductible did, therefore, not result in a financial benefit for patients with annual costs exceeding their total deductible.

## Introduction

Managed care insurers that are more successful at channeling patients can negotiate better deals with health care providers [7, 19, 26]. A credible threat of losing patient volume seems to stimulate providers to offer more favorable contract terms, such as price discounts and quality improvements, than they otherwise would have offered. To channel patients to specific providers, insurers may differentiate cost-sharing rates across provider alternatives requiring higher out-of-pocket payments for visits to non-preferred providers than for visits to preferred providers. There is emerging empirical evidence from the US that the use of financial incentives affects patient choice behavior (see for example [5, 16, 18]). These incentives include differential copayments across provider tiers, percentage coinsurance rates which automatically tiers providers according to price, and reimbursement limits requiring the patient to pay the difference between this limit and the insurer–provider negotiated price.

This paper examines a channeling experiment with a differential deductible in The Netherlands. In this 1-year experiment De Friesland Zorgverzekeraar (DFZ), at that time the largest independent regional health insurer in The Netherlands, designated preferred providers for two procedures (cataract surgery and varicose veins treatment). It also used a financial incentive to encourage their enrollees to choose these providers for medical treatment. The insurer exempted its enrollees from paying their annual deductible when they sought care at a preferred provider. The deductible exemption, however, was conditional on the enrollee’s other medical expenses occurring in the policy year. People still had to pay their annual deductible when using other medical services than cataract surgery and varicose veins treatment. During the experiment, the enrollee’s financial benefit when visiting a preferred provider thus depended upon his ex post total medical expenses in the policy year. Hence, people had to make a prediction about the ‘price’ associated with visiting a non-preferred provider.

To test whether this channeling experiment affected the allocation of patients across individual providers, Van der Geest and Varkevisser [21] estimated two OLS regression models using providers’ patient volume data over a 3-year period.^1^ In this study, it was concluded that in the year of the experiment the allocation of cataract patients across individual providers was not affected by the channeling experiment, whereas preferred providers of varicose veins treatment on average gained patient volume relative to non-preferred providers. Both the insurer’s selection of preferred providers in terms of joint market share and the design of the financial incentive are likely explanations for this result.

As a follow-up study, this paper focuses on the procedure for which the allocation of patients across providers was significantly affected. It assesses in more detail how responsive patients were to the cost-sharing differential between preferred and non-preferred providers of varicose veins treatment apart from the insurer’s preferred provider label. It contributes to the emerging, but still small body of literature examining the effect of differential cost-sharing on patient provider choices. Using additionally obtained data on each individual patient’s out-of-pocket payment and a few patient characteristics, we estimate a conditional logit model of patient choice to empirically disentangle the effect of the preferred provider label and the financial channeling incentive provided. We use choice data covering the year of the experiment and 1 year before to control for pre-existing patient preferences. The estimated coefficients are then used for calculating preferred and non-preferred providers’ average cross-price elasticity of demand.

The paper is structured as follows. We first briefly summarize the institutional context and the natural experiment. Then we describe our empirical methodology, followed by a discussion of the data. After this, we present our estimation and simulation results. We conclude with a summary and discussion of our main findings.

## Background

The Netherlands has a system of universal health insurance based on regulated competition in the private sector. This system adheres closely to Enthoven’s plan of managed competition in health care [4]. All citizens are obliged to buy standardized basic health insurance covering the costs of all common medical care including primary care, hospital services (for up to 1 year), and pharmaceuticals. The premium for basic health insurance is community-rated. There is a risk equalization system in place to reduce insurers’ incentives for risk selection. Every adult has a mandatory annual deductible that must be met before medical services are reimbursed by the insurer (excluding primary care and maternity care). Consumers obtain a community-rated discount on their premium if they opt for a voluntary deductible (at most 500 euro).

Competing private health insurers are provided with financial incentives as well as tools to organize and manage acute (curative) care for their enrollees by establishing and maintaining provider networks. To increase their bargaining power vis-à-vis providers, health insurers may restrict access to a network of providers. Enrollees using out-of-network providers receive a lower reimbursement rate. Other than selective contracting, insurers can designate preferred providers within their contracted network, i.e. forming a two-tiered provider network. To encourage enrollees to choose providers labelled as ‘preferred’ or ‘higher value’ they may waive the annual deductible for visiting a preferred provider.^2^

DFZ, then the largest regional health insurer in The Netherlands with a market share of about 65% in the Dutch province Friesland (or Frisia),^3^ used such a differential deductible for patient channeling in 2009. It designated three hospitals (including the largest hospital in Friesland) and one freestanding ambulatory surgery center as preferred providers for varicose veins treatment because of above average performance in guideline adherence, waiting time and patient satisfaction.^4^ To enrollees it was emphasized that the preferred providers were carefully selected for reasons of quality and, therefore, labelled as ‘higher value’. The deductible exemption would concern both the enrollee’s mandatory deductible (155 euro in 2009) as well as, when relevant, the voluntary deductible (at most 500 euro). The difference in cost-sharing between preferred and non-preferred providers could, therefore, add up to a maximum of 655 euro. Hospital-insurer negotiated prices for the treatment of varicose veins typically differed from about 700 euros to about 2000 euros for the more complex treatments and thus in 2009 exceeded the patient’s deductible.^5^ However, the deductible exemption was conditional on the enrollee’s other medical expenses occurring in the year 2009. Enrollees still had to pay their annual deductible when using other medical services than varicose veins treatment and cataract surgery.^6^

## Empirical methodology

The aim of our study was to assess in detail how responsive patients were to the insurer’s preferred provider label and the cost-sharing differential between preferred and non-preferred providers. We model patient provider choice using a utility maximization framework in which the patient chooses the provider that is most attractive based on attributes that vary across alternative providers.^7^ To estimate our model we use pooled individual patient choice data from 1 year before (2008) and during the channeling experiment (2009). This allows us to control for unobservable prior preferences for providers that were designated as preferred provider by the insurer in 2009, as it is very unlikely that patient preferences (in terms of trade-offs between provider attributes) changed simultaneously with the introduction of the patient channeling experiment in these two consecutive years. Since all DFZ insured in 2009 were exposed to the financial incentive, we are not able to analyze whether the pre-to-post-difference was larger for exposed patients than for non-exposed patients. Our control group, therefore, necessarily consists of all varicose veins patients in 2008 since they were not exposed to any channeling incentive. Thus, we compare the probability that a given provider would be chosen by patients if that provider was selected as a preferred provider in 2009 with the probability that it was chosen in the year prior to the experiment.

As is standard in the contemporary hospital choice literature (see for example [1, 3, 5, 6, 8, 10], we use the random utility choice model introduced by McFadden [14]. Assuming the unobserved random components (*ε*_*ij*_) are independently and identically distributed (idd), we estimate the following conditional logit model specification for analyzing patient choice:^8^

1 $${\text{Choic}}{{\text{e}}_{ij}}={\beta _1}{\text{Pos}}{{\text{t}}_i}+{\beta _2}{\text{Pre}}{{\text{f}}_j}+{\beta _3}{\text{Pos}}{{\text{t}}_i}*{\text{Pre}}{{\text{f}}_j}+{\beta _4}{\text{Pric}}{{\text{e}}_{ij}}+{\beta _5}{\text{Tim}}{{\text{e}}_{ij}}+\mathop \sum \limits_{{k=1}}^{n} {\gamma _k}{H_{kj}}+{\varepsilon _{ij~}}$$

where Choice_*ij*_ is a dummy variable identifying patient’s *i* choice of hospital *j* given all *J* hospitals in his choice set. Post_*i*_ is a dummy variable equal to 1 if patient *i*’s varicose veins treatment took place in 2009 when the two-tiered provider network was in place (and 0 if treatment took place in 2008). Pref_*j*_ is a dummy variable indicating whether provider *j* belongs to the group preferred providers. It controls for pre-existing patient preferences for the providers designated as preferred provider by DFZ in 2009. The coefficient on the interaction between Post_*i*_ and Pref_*j*_ represents the change in utility from choosing a preferred provider in the year of the experiment. This is thus one of the coefficients of our primary interest while it allows us to test whether the preferred provider status affected patient choice behavior. Please note that *β*_1_Post_*i*_ will drop out the regression, as it does not vary within the choice set.

Through the price component of the utility function, we test whether the financial channeling incentive affected patient choice behavior. The variable Price_*ij*_ is the financial benefit (in euros) that patient *i* would miss out when visiting a non-preferred provider. Of course, this euro amount was equal to zero for all non-preferred providers in 2008, because at the time there was no financial incentive in place yet. However, during the experiment the price of non-preferred providers was based on patient’s *i* expectation regarding the amount of his total deductible left unused in the 2009 policy year. In the absence of more detailed information about patients’ total health care consumption, we use each patient’s percentage of the total deductible left unused in 2008 multiplied by the total deductible amount (i.e. sum of mandatory and voluntary deductible) in 2009 as a proxy for his expected financial benefit.^9^ This approach is similar to how Brot-Goldberg et al. [2] model a consumer’s expected end-of-the-year marginal price. Unfortunately, the obtained data do not allow us to control in a meaningful way for the fact that patients may have adjusted their expectations during the year of the experiment.^10^ Note that using each patient’s percentage of the deductible left unused in the year of the experiment as an alternative would incorrectly assume perfect foresight and also result in an endogeneity problem.

The variable Time_*ij*_ is the minimum driving time from the patient *i*’s home to provider *j*. The vector ${H_{ \cdot j}}$ represents two provider attributes we control for; i.e. provider type (general hospital, tertiary hospital or ambulatory surgery center) and whether the provider is located in the province of Friesland.^11^ Clinical quality as provider attribute is not included in the model as choice determinant because public information about it was unavailable during the study period. Reliable outcome indicators for varicose veins treatment were being developed in The Netherlands at the time, but not yet available for patients (and GPs) to compare providers [25]. Note that differences in waiting time across providers are implicit part of the model because it was one of indicators used by the insurer when selecting the preferred providers. The error term (*ε*_*ij*_) represents the idiosyncratic part of patient *i*’s evaluation of provider *j* including information obtained by word of mouth and possible prior experience.

To test whether there is patient heterogeneity in responsiveness to the channeling experiment, we also estimate an extended version of Eq. [1] where we interact both Post_*i*_ × Pref_*j*_ and Price_*ij*_ with three patient characteristics: age, gender and social status. For age we construct a dummy variable to define the subgroup of retired people (in 2009 the standard retirement age was 65 years) who usually experience a (substantial) drop in income after retirement.

## Data

### Data sources

The data for this study come from multiple sources. Most importantly, from health insurer DFZ we obtained individual claims data for all their varicose veins patients capturing the period January 2008 through December 2009. This data include the Diagnosis and Treatment Combination (DTC) code of the specific varicose veins treatment only,^12^ the date of admission, the provider name and postal code, the patient’s gender, age (on date of admission) and his residential postal code. For patients admitted in 2009, the insurer also provided information on the amount of voluntary deductible chosen for the calendar years 2008 and 2009 as well as the euro amount paid to the insurer of his total deductible (i.e. sum of mandatory and any voluntary deductible) in 2008 and 2009.^13^ For the 2009 patient group we calculate the percentage left unused of the total deductible in 2008 to construct our proxy variable for the financial channeling incentive.

We used a drive time matrix containing all 4-digit postal codes in The Netherlands to include the minimum driving time (in minutes) from an individual’s residential postal code to each provider.^14^ Data on type of provider are obtained from the web-based Dutch National Atlas of Public Health and the website of the Dutch association of tertiary medical teaching hospitals. Based on the provider’s postal code, we construct the dummy variable whether a provider is located in Friesland or not.

Due to privacy concerns, DFZ could not provide any detailed patient-level socio-economic information. In addition to the available individual patient data on age and gender, from The Netherlands Institute for Social Research (SCP) we obtained a social status score for each 4-digit postal code in 2010. Using factor analysis, SCP derived the social status of a 4-digit postal code area from a number of characteristics of the people living there: average income level, the percentage of people having a low income, the percentage low educated and the percentage unemployed. The higher the score, the higher the social status of the postal code area.

### Patient study sample and patients’ choice sets of providers

For the analysis, we use the obtained claims data to create a study sample of varicose veins patients. To prevent a bias caused by existing patient–physician relationships we only include for each patient the first primary treatment of the calendar year.^15^ In addition, we selected patients living in Friesland because the patient channeling experiment was primarily aimed at this population. From all DFZ enrollees that needed treatment for varicose veins in 2008–2009 as much as 85% lived in Friesland. The other 15% were almost equally spread across the country.

For some patients the name of the provider visited is missing in the data, so we exclude them from the analysis (*n* = 69). We also drop all patients who were treated by a provider not located in the northern part of the country (*n* = 28). Since this small minority of patients travelled on average much longer than other patients (i.e. 119 min compared to 25 min), it is most likely that either these patients’ starting addresses are incorrect or their observed provider choice reflects very special preferences or medical needs.

**Fig. 1:**
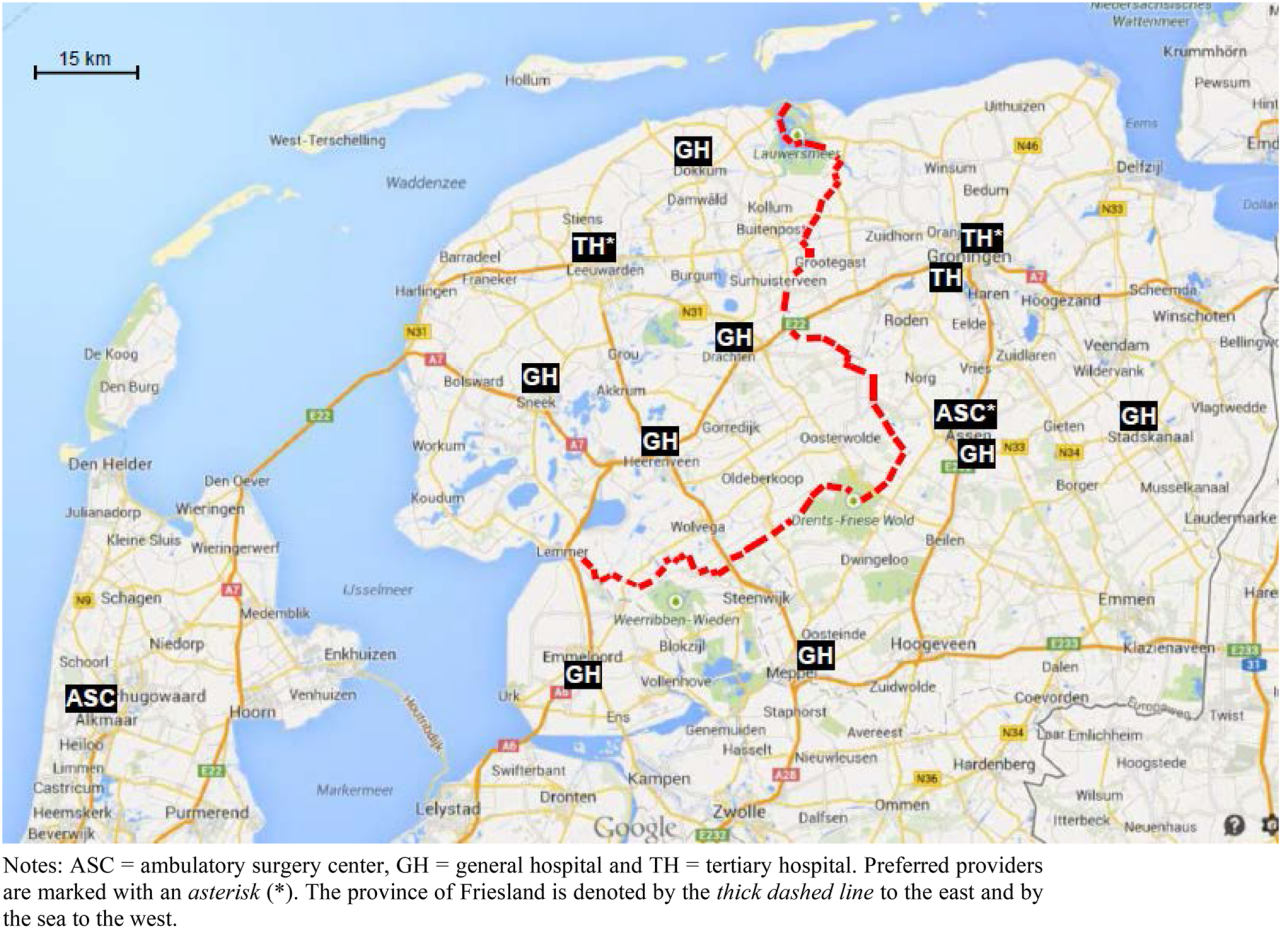
Location of providers included in the study

As it is not possible to observe a patient’s true choice set in these data, we rely on the aggregated choices of patients to identify supposed reasonable options. We presume each patient’s choice set to consist of all providers contracted by DFZ for varicose veins treatment (located in the northern part of the country) that were visited by at least one enrollee in each sample year. This results in a uniform choice set for each patient including 13 different providers. Consequently, an additional small number of patients (*n* = 78) is excluded, because they visited a provider that was outside this choice set. In the choice set of 2009 three providers were designated as preferred provider.^16^ The location of all providers included in the choice set is shown in Fig. 1. More detailed information on these providers can be found in the “Appendix” (Table 6).

## Results

### Descriptive statistics

Our final study sample includes 4252 unique varicose veins patients with DFZ insurance during the study period 2008–2009. Table 1 presents the descriptive statistics. From this table it follows that the 2008 and 2009 patient groups are quite similar. Almost 80% of the patients were females and patients were on average slightly older than 50 years.^17^ The average social status scores of the 2008 and 2009 patient group were − 0.54 and − 0.48, respectively, which is below the national average score (0.17). On average, patients travelled less than 25 min and almost all patients (95 and 93% in 2008 and 2009, respectively) did not leave the Frisian province for treatment. About 60% of the patients visited a general hospital, while around 35% visited a tertiary hospital. A considerable lower percentage of patients (3–6%) obtained care at an ambulatory surgery center. In 2008, the three providers designated preferred during the channeling experiment jointly performed 39% of the procedures. The largest hospital in the region (Medisch Centrum Leeuwarden) represented 91% of this volume. In 2009, the preferred providers jointly performed 42% of the procedures. In both absolute and relative terms (49 patients and 71%, respectively) the freestanding ambulatory center among the preferred providers (Braamkliniek) experienced the biggest increase in patient volume.

**Table 1:** Descriptive statistics of patient study sample

	2008 *n* = 2123				2009 *n* = 2129			
	Mean	Std. dev	Min	Max	Mean	Std. dev	Min	Max
Age (years)	51	14	15	87	51	14	21	90
Social status	− 0.54	1.17	− 7.25	2.94	− 0.48	1.13	− 5.49	3.19
Travel time (minutes)	24	39	0	455	25	39	0	455
Female	0.78		0	1	0.77		0	1
Age65+	0.19		0	1	0.18		0	1
Located in Friesland	0.95		0	1	0.93		0	1
Type of provider								
ASC	0.03		0	1	0.06		0	1
General hospital	0.61		0	1	0.57		0	1
Tertiary hospital	0.36		0	1	0.37		0	1
Preferred provider	0.39^a^		0	1	0.42		0	1

^a^Proportion of patients in 2008 visiting a provider that would be designated as preferred provider in 2009

Table 2 presents some additional descriptive statistics of the 2009 patient group. The average total deductible—and, therefore, the maximum financial benefit for the average patient—in 2009 was 159.46 euro which is only slightly higher than the mandatory deductible in that year. The number of patients who opted for a voluntary deductible is in fact negligible (less than 2%). On average, the patients in the 2009 study sample left 22% of the total deductible unused in the year prior to the experiment resulting in an average *expected* financial benefit of 36.84 euro that one would miss out when visiting a non-preferred provider. However, there is significant variation across individual patients.

**Table 2:** Descriptive statistics of 2009 patient study sample

	2009 *n* = 2129			
	Mean	Std. dev	Min	Max
Mandatory deductible in 2009 (euros)	155.00	0.00	155.00	155.00
Voluntary deductible in 2009 (euros)	4.46	39.54	0.00	500.00
Total deductible in 2009 (euros)	159.46	39.54	155.00	655.00
Deductible left unused in 2008 (%)	22	36	0	100
Financial benefit of preferred provider choice (euros)	36.84	71.30	0	655.00

### Model specifications

We estimate two model specifications: model A and model B. In addition to preferred provider status and price, the explanatory variables in both models include travel time, located in Friesland and type of provider to capture dimensions of provider heterogeneity that may have affected patient provider choice. The difference between both models is that model B allows both the effect of designating preferred providers and the financial channeling incentive to be heterogeneous among patient groups. These groups are identified using the few patient characteristics available in the dataset.

### Goodness of fit

To measure the models’ goodness of fit, following Town and Vistnes [20], we construct a “hit-or-miss” criterion where predicted patient choice was the provider with the maximum predicted probability. Both model A and B correctly predict almost 8 out of every 10 patients’ provider choices, suggesting a high degree of explanatory power. In addition, we predict each provider *j*’s patient volume by summing up all patients’ estimated choice probabilities for provider *j*. Table 7 in the “Appendix” presents predicted patient volume for each individual provider using the two models. Notice that from this table it can be concluded that one of the preferred providers (UMC Groningen) apparently was not an attractive alternative for the patients included in the study sample; most likely because a university medical center typically focuses on top clinical and highly specialized care.

### Estimated coefficients

Table 3 reports our patient choice model’s coefficient estimates and standard errors. Consistent with the existing empirical literature on patient provider choice, we find that varicose veins patients had a strong preference for providers located nearby, all else equal. In both models, the coefficient of Time is highly significant and negative. An artificial 10% increase in travel time, all else equal, reduces a provider’s patient volume between 11 and 84% as minimum and maximum respectively. Related to this, patients also had a high propensity of selecting a provider located in the province Friesland, all else equal. Hence, they did not like to cross the regional border for treatment. Furthermore, patients were more likely to choose ambulatory surgery centers compared with general and tertiary hospitals, all else equal.

**Table 3:** Conditional logit estimates of patient choice

Variable	Model A			Model B		
	*β*	Sig.	SE	*β*	Sig.	SE
Pref	0.612		0.341	0.610		0.341
Post × Pref	0.219	**	0.094	− 0.080		0.174
Post × Pref × Age65+				− 0.217		0.187
Post × Pref × Female				0.487	***	0.174
Post × Pref × Social status				0.139	**	0.064
Price	− 0.002	**	0.001	− 0.000		0.001
Price × Age 65 +				− 0.009	**	0.003
Price × Female				− 0.001		0.002
Price × Social status				0.000		0.001
Travel time	− 0.128	***	0.002	− 0.128	***	0.002
Located in Friesland	1.676	***	0.149	1.669	***	0.149
Tertiary hospital	− 0.246		0.337	− 0.249		0.338
ASC	3.580	***	0.424	3.617	***	0.424
*N* observations	55,276			55,276		
*N* patients	4252			4252		
Correct predicted (%)	79			79		

**Significant at the 5% level

***Significant at the 1% level

In model A the significant coefficient of Pref interacted with Post indicates that being designated as preferred provider by health insurer DFZ in 2009 was a positive incentive for patients (*p* value = 0.02). Patients were more likely to visit those providers, all else equal. The coefficient of Pref is not statistically significant (*p* value = 0.07). Hence, it is not particularly apparent that patients had a pre-existing preference for providers that were designated as preferred provider in 2009. The negative sign found for the significant coefficient on Price in model A indicates that the financial channeling incentive affected patient provider choice in the expected direction (*p* value = 0.04). The higher the price patients were expected to pay out-of-pocket, the less likely they were to seek care from a non-preferred provider. However, in contrast to the other provider attributes, patients were relatively insensitive to the financial benefit of visiting a preferred provider.

Despite the absence of an overall effect (i.e. for the “average” patient), the interaction variables in model B—capturing any differences in preferences across types of patients—suggest a statistically significant effect for females and social status with respect to the effect of the preferred provider status on patient choice.^18^ The estimates indicate that females were more responsive to the preferred provider status than males. The same seems to hold for patients with a higher social status, which might reflect a higher cognitive ability to understand the channeling instrument. It might also relate to challenges faced by patients with a lower social status. For example, a reliance on public transportation would make travelling to a distant preferred provider more difficult for them. The significant coefficient on Price interacted with Age65+ suggests that retired patients were more sensitive to the financial channeling incentive than their younger counterparts (*p* value = 0.01). It looks like the retired patient group—or the relatively healthy subpopulation in this group—took notice of the provided channeling incentive as well as expected themselves, based on their out-of-pocket payment in 2008, to benefit financially when choosing one of the preferred providers in 2009.

### Cross-price elasticity of demand

We conduct a simulation analysis for examining patients’ responsiveness to hypothetical changes in the cost-sharing rate.^19^ Using model B we simulate the impact of a twofold increase in patients’ 2009 total deductible on patient volume. Following our proxy for patient’s *i* expectations about his (potential) financial benefit, a twofold higher total deductible equals a doubling of the patient’s maximum price of a non-preferred provider. For the average patient this corresponds to a maximum financial benefit of about 320 euro. Using the conditional logit estimates from Table 3, we recalculated each patient’s choice probabilities for all individual providers when the hypothetical increase in deductible would apply. After summing up these probabilities at the provider level, the percentage change in predicted patient volume for the preferred providers is divided by the percentage change of the deductible. Table 4 presents the results of this simulation analysis. Based on the estimated coefficients, the hypothesized larger cost-sharing difference across preferred and non-preferred providers would increase the predicted number of patients visiting the three preferred providers by 2.4 percent. Hence, the mean cross-price elasticity of demand equals 0.02 ( SE = 0.00).^20^ As a robustness check, we also performed the simulation analysis in two alternative ways. First, for the three patient subgroups separately (retired, female, lowest social status) the mean cross-price elasticities are found to be 0.05 (*n* = 153), 0.02 (*n* = 727), and 0.02 (*n* = 537), respectively. So, as follows from model B’s coefficients, the retired patient group seems slightly more responsive to the channeling instrument. Second, when using model A rather than model B the mean cross-price elasticity of demand also equals 0.02 (*n* = 899). Hence, including interaction terms does not change this elasticity.

**Table 4:** Estimated cross-price elasticity of demand (price of non-preferred providers + 100%)

Provider	Predicted patient volume	Mean	SE	95% confidence based on parametric bootstrap^a^	
MC Leeuwarden	795	0.02	0.000	0.02	0.02
Braamkliniek	98	0.03	0.000	0.02	0.03
UMC Groningen	6	0.07	0.001	0.06	0.07
Preferred providers	899	0.02	0.000	0.02	0.02

^a^For the parametric bootstrap we use the Krinsky–Robb method [12, 13]

### Who really benefited?

Another look at the data confirms the limited impact of the cost-sharing differential itself. In 2009, from the 2129 patients 42% chose one of the preferred providers. Based on their out-of-pockets payments in 2008, a total of 655 patients (31%) was expected to benefit from the difference in cost-sharing across the two tiers of providers (Table 5). This group includes all patients who did not fully use their deductible in the year preceding the channeling experiment. From them, 274 patients (42%) indeed selected a preferred provider for their treatment in 2009. The remaining 381 patients (58%), for whatever reason, during the channeling experiment did not respond to their prior end-of-the-year marginal price.

**Table 5:** Number of patients by provider choice in 2009 and expected financial benefit of preferred provider choice

Provider choice in 2009	Expected financial benefit:		Total
	> €0–€155	> €155–€655	
Preferred provider	258	16	274
Non-preferred provider	370	11	381
Total	628	27	655

Using the data that DFZ provided us on out-of-pocket payments in 2009, additional calculations reveal that only 19% of the 899 patients who during the channeling experiment selected a preferred provider did actually enjoy a financial benefit. For them this benefit was on average almost 100 euro. For the remaining patients the costs of medical services other than treatment for varicose veins were so high that they still had to pay their full deductible. Hence, the overwhelming majority of patients (81%) choosing to visit a preferred provider at the end of the year was not financially rewarded for doing so.

## Conclusion

Health insurers offering managed care plans may use financial incentives for channeling patients to preferred providers with lower costs and/or higher quality. Empirical evidence whether differences in cost-sharing rates across providers impacts patient choice behavior is emerging but, especially from European countries, still limited. This paper examined a Dutch insurer’s channeling experiment with a differential deductible to steer patient provider choice in a regional market for varicose veins treatment. During the experiment, the insurer waived patients’ deductible when they chose to visit a preferred provider for medical treatment. Since the exemption was conditional on the patient’s other medical expenses occurring in the policy year, people had to make a prediction about the ‘price’ associated with visiting a non-preferred provider.

Using data covering the year of the experiment and 1 year before, we estimated a conditional logit model of patient choice. Our main results can be summarized as follows.

First, the estimation results indicate that, independent of their expected financial benefit imposed by the differential deductible, patients were more likely to choose a preferred provider than a non-preferred provider. In the year preceding the experiment, a clear preference for these providers was not observed. This suggests that the insurer succeeded in convincing a considerable number of patients of the preferred providers’ better than average performances on guideline adherence, waiting time and patient satisfaction.

Second, the estimation results suggest that varicose veins patients were less likely to select a non-preferred provider when this, based om their percentage use of deductible in 2008, was associated with a higher expected out-of-pocket payment. The average cross-price elasticity of demand is estimated to be 0.02, indicating that patient responsiveness to the cost-sharing differential itself was low.^21^

To conclude, our finding that patient demand for the preferred providers is rather insensitive to an increase in price of the non-preferred providers may be related to the relatively short length of the experiment. By canceling it already after the first year, the insurer ruled out the possibility that more enrollees learned about the financial incentive in later years. The channeling experiment lasted only 1 year because, according to the insurer, enrollees reacted negatively towards the use of a differential deductible to influence patient choice. Furthermore, it seems likely that the design of the financial incentive contributed substantially to a low patient responsiveness. Unlike a fixed cost-sharing differential between preferred and non-preferred providers the deductible exemption studied here was conditional on the patient’s other medical expenses occurring in the policy year. Most patients were, therefore, uncertain about their financial benefit of choosing a preferred provider making *ex ante* price comparison very difficult. On the other hand, the financial incentive was irrelevant for chronically ill patients because they will for sure meet their deductible within the year and thus do not have any incentive to respond to this channeling experiment.

Reducing the annual deductible when enrollees seek care at a preferred provider instead of waiving it only for that treatment might be a more effective financial incentive for patient channeling. However, it would also be more costly to insurers and causing a potential moral hazard effect on the use of other medical services. Moreover, a first prerequisite for a differential deductible to work as intended is that patients understand the concept of a deductible compared to their amount of medical spending. This is, as clearly illustrated by Handel and Kolstad [9], not straightforward. When attempting to steer patients to preferred providers, we, therefore, expect insurers to be more successful using other types of cost-sharing differentials by provider tier, for example, making a cost-sharing distinction for co-payments, co-insurance or the out-of-pocket maximum. This provides patients with a financial incentive that is both easy to understand and associated with a guaranteed financial benefit.

## Appendix

See Tables 6 and 7.

**Table 6:** Provider attributes

	Provider name	City	Province	Type^a^	Preferred provider
1	MC Leeuwarden	Leeuwarden	Friesland	TH	Yes
2	Ziekenhuis Nij Smellinghe	Drachten	Friesland	GH	No
3	Antonius ziekenhuis	Sneek	Friesland	GH	No
4	Ziekenhuis De Tjongerschans	Heerenveen	Friesland	GH	No
5	Talma Sionsberg	Dokkum	Friesland	GH	No
6	Braamkliniek	Assen	Drenthe	ASC	Yes
7	Wilhelmina ziekenhuis	Assen	Drenthe	GH	No
8	UMC Groningen	Groningen	Groningen	TH	Yes
9	IJsselmeer ziekenhuis	Noordoostpolder	Flevoland	GH	No
10	Flebologisch centrum Oosterwal	Alkmaar	N-Holland	ASC	No
11	Martini ziekenhuis	Groningen	Groningen	TH	No
12	Refaja ziekenhuis	Stadskanaal	Groningen	GH	No
13	Zorgcombinatie Noorderboog	Meppel	Drenthe	GH	No

^a^*ASC* ambulatory surgery center, *GH* general hospital, *TH* tertiary hospital

**Table 7:** Actual and predicted patient volumes

	Provider name	Actual patient volume		Predicted patient volume									
				Model A					Model B				
		2008	2009	2008	2009	% total in 2008	%total in 2009	Diff. (%)	2008	2009	% total in 2008	% total in 2009	Diff. (%)
**1**	**MC** **Leeuwarden**	**747**	**771**	**726**	**794**	**34.2**	**37.3**	**3.1**	**725**	**795**	**34.1**	**37.3**	**3.2**
2	Ziekenhuis Nij Smellinghe	397	385	361	350	17.0	16.4	− 0.6	361	350	17.0	16.4	− 0.6
3	Antonius ziekenhuis	415	415	403	360	19.0	16.9	− 2.1	403	361	19.0	17.0	− 2.0
4	Ziekenhuis De Tjongerschans	333	252	315	272	14.8	12.8	− 2.1	315	271	14.8	12.7	− 2.1
5	Talma Sionsberg	127	156	191	225	9.0	10.6	1.6	191	226	9.0	10.6	1.6
**6**	**Braamkliniek**	**69**	**118**	**93**	**99**	**4.4**	**4.7**	**0.3**	**94**	**98**	**4.4**	**4.6**	**0.2**
7	Wilhelmina ziekenhuis	13	11	2	2	0.1	0.1	0.0	2	2	0.1	0.1	0.0
**8**	**UMC Groningen**	**8**	**10**	**5**	**6**	**0.2**	**0.3**	**0.0**	**5**	**6**	**0.2**	**0.3**	**0.0**
9	IJsselmeer ziekenhuis	5	2	9	8	0.4	0.4	0.0	9	8	0.4	0.4	0.0
10	Flebologisch centrum Oosterwal	4	6	3	1	0.1	0.0	− 0.1	4	1	0.2	0.0	− 0.1
11	Martini ziekenhuis	3	1	5	4	0.2	0.2	0.0	5	4	0.2	0.2	0.0
12	Refaja ziekenhuis	1	1	0	0	0.0	0.0	0.0	0	0	0.0	0.0	0.0
13	Zorgcombinatie Noorderboog	1	1	9	6	0.4	0.3	− 0.1	9	6	0.4	0.3	− 0.1
	Total	2123	2129	2123	2129	100	100		2123	2129	100	100	
	Total of preferred providers	824	899	824	899	38.8	42.2	3.4	824	899	38.8	42.2	3.4
	Total of non-preferred providers	1299	1230	1298	1228	61.1	57.7	− 3.5	1299	1229	61.2	57.7	− 3.5

The preferred providers are highlighted in bold

## Footnotes

1 The data was obtained from health insurer DFZ. Hence, each provider’s patient volume is the number of patients with health insurance provided by DFZ.

2 For more information about the bargaining position of Dutch health insurers in the negotiations with health care providers, see Schut & Varkevisser [17].

3 Friesland is a province in the northwest of The Netherlands. Most of Friesland is on the mainland, but it also includes four small islands which are connected to the mainland by ferry. The total land area equals 3349 km^2^. Friesland has a total population of 646,000 and is with a population density of 190 inhabitants per km^2^ one of the most sparsely populated regions in The Netherlands.

4 Varicose veins are swollen and enlarged veins that are visible through the skin and may appear blue or dark purple. They are caused by increased blood pressure inside the superficial veins and commonly develop in the legs and ankles. Besides being a cosmetic problem, varicose veins can be very painful. Women are more likely to be affected by varicose veins than men. Treatment for varicose veins performed by medical specialists requires a referral from a general practitioner (GP) and involves both surgical (e.g. vein stripping) and nonsurgical approaches (e.g. compression stockings, sclerotherapy and laser treatment). The vast majority of patients is treated in an outpatient setting.

5 For confidentiality reasons, DFZ did not provide us with information on their negotiated prices.

6 The actual financial benefit of choosing a preferred provider has been different among patient groups. Patients with no additional medical expenses in 2009 had a higher financial benefit of choosing a preferred provider than other patients. Furthermore, enrollees who opted for a voluntary deductible in 2009 had a higher potential financial benefit than enrollees with no voluntary deductible.

7 In The Netherlands, GPs function as gatekeepers for specialized medical care but patients are free to choose which provider to visit. Based on videotaped Dutch GP-patient consultations, Victoor et al. [24] concluded that many patients visit the provider that is recommended by their GP. In the study period, Dutch GPs did not have a (financial) interest to refer patients to particular providers while neglecting their preferences. We therefore do not take into account potential principal-agent problems. The term patient here thus refers to the patient-referring GP pair jointly choosing the most attractive provider. Note that the patient’s (potential) financial benefit associated with the choice of a preferred provider may not be the deciding factor.

8 Time subscripts are not included in the model specification, because we pooled the data from 2008 to 2009. The variable *Post*_*i*_ identifies whether an observation is either from before or after the start of the channeling experiment.

9 For 99.4 percent of the patients in our study sample the total deductible in 2009 was the same as in 2008, except for the increase of the mandatory deductible imposed by the government (+ 5 euro).

10 Because we cannot track patients across claims related to other types of medical care other than varicose veins treatment, it is not possible to control for the presence of chronic conditions. Hence, we are not able to distinguish chronically ill from non-chronically ill patients who likely differed on expectations regarding their medical expenses in 2009.

11 Other things being equal, Frisians might prefer regional providers because they are generally speaking proud of their own culture and identity. As an illustration, Friesland is the only one of the twelve provinces of The Netherlands to have, in addition to Dutch, its own official language.

12 In The Netherlands each patient admitted to a provider of specialized medical care is categorized into a Diagnosis and Treatment Combination (DTC) code, which includes all outpatient and/or inpatient activities and services associated with the patient’s care from the initial consultation to the final check-up.

13 For only a very few patients (*n* = 27) DFZ could not provide us with data on out-of-pocket payment and amount of voluntary deductible in 2008 because they were not enrolled in that year.

14 The drive time matrix also accounts for differences in average speed that exist between different road types.

15 If there are varicose veins in both legs that require treatment, these treatments are almost always registered as separate claims and performed on different dates. Nearly one-third of the patients in our sample required more than one treatment during a calendar year. In these cases, switching provider for the next treatment is rare (less than 5 percent in our sample).

16 The fourth provider designated as preferred provider by DFZ, a general hospital located relatively far away, was not included because none of the Frisian patients with DFZ insurance visited this provider in the study period.

17 Compared to the overall Dutch population females and elderly are overrepresented among the varicose veins patients.

18 Please note that when included in non-linear models the coefficients of interaction terms must be interpreted carefully [11].

19 This approach is commonly used in the hospital choice literature; e.g. Varkevisser et al. [22, 23], Beukers et al. [1], and Gutacker et al. [8].

20 The standard error is computed to test for the robustness of our findings. We used the vector of estimated parameters and corresponding covariance matrix from our conditional logit model to randomly draw 1000 sets of alternative, equally probable model parameters from a multivariate normal distribution with this vector and matrix as means and covariance, respectively.

21 As pointed out by one of the reviewers, our study does not take into account that some patients requiring varicose vein treatment may forego treatment or switch to a different treatment rather than receive a (potentially) higher-cost procedure from a non-preferred provider. In theory, such substitution might indeed be relevant for patients who would choose to receive care from a non-preferred provider absent the tiering scheme. However, in practice the deductible—which underlies the potential price difference between preferred and non-preferred providers—is in The Netherlands not associated with unmet need for financial reasons [15].
